# Isolation and characterization of *Listeria monocytogenes* from environmental and clinical sources by culture and PCR-RFLP methods

**Published:** 2019-02

**Authors:** Hossein Meghdadi, Azar Dokht Khosravi, Ahmad Farajzadeh Sheikh, Ameneh Alami, Nerssy Nassirabady

**Affiliations:** 1 Department of Microbiology, Faculty of Medicine, Ahvaz Jundishapur University of Medical Sciences, Ahvaz, Iran; 2 Infectious and Tropical Diseases Research Center, Health Research Institute, Ahvaz Jundishapur University of Medical Sciences, Ahvaz, Iran; 3 Cell and Molecular Research Center, Ahvaz Jundishapur University of Medical Sciences, Ahvaz, Iran

**Keywords:** *Listeria monocytogenes*, Genotyping, Restriction enzyme, Polymorphism

## Abstract

**Background and Objectives:**

Due to the widespread distribution of *Listeria monocytogenes* in environmental and animal sources and serious clinical complications in human, this study was aimed to isolate *L. monocytogenes* from water and clinical specimens by culture and PCR methods and to investigate the presence of *hlyA* and *inlA* virulence genes.

**Materials and Methods:**

Water and clinical samples of vaginal and fecal were screened for the presence of *L. monocytogenes* by phenotypic and standard biochemical tests. PCR amplification was performed on extracted DNA using primers based on the *hlyA* and *inlA* genes. A 733-bp fragment of *inlA* gene was used for investigation of polymorphism using RFLP analysis.

**Results:**

In total, 45 phenotypically and molecularly confirmed *L. monocytogenes* strains were isolated from different sources including 30 (16.7%) from water, 9 (11.3%) from vaginal swabs and 6 (7.5%) from fecal samples. RFLP analysis of PCR products using *AluI* and *Tsp509I* restriction enzymes, generated two profiles with 8 to 10 bands ranging in size from 15 to 210 bp. The majority of water and clinical isolates were classified in profile 2.

**Conclusion:**

We demonstrated 45 *L. monocytogenes* isolates from tested water and clinical samples by phenotypic and molecular tests. The majority of the isolates were classified in the same RFLP profile, showing the water as a potential source of clinical complications in patients in the region of study.

## INTRODUCTION

*Listeria monocytogenes* is a Gram-positive, non-sporulating, intracellular bacillus, with ubiquitous presence in land and water environments, feces, vegetables and gastrointestinal tracts (1, 2). The organism was first described by Murray et al., and named “*Bacterium monocytogenes*”, due to the characteristic of monocytosis in infected laboratory animals, and was renamed “*Listerella monocytogenes*” by Pirie in 1940 (3, 4). Due to its psychrophilic nature, it can grow within a wide range of temperatures (−1.5 to 50°C), and therefore grows easily in the water ecosystem (2). Additionally, municipal sewage sludge which is spread onto agricultural lands, contains many pathogens including *L. monocytogenes*, therefore, agricultural products and ultimately livestock products are contaminated with this organism (5).

The presence of *L. monocytogenes* in water may be a cause of listeriosis in human and animals (6). The microorganism is causing fatal infections such as encephalitis, sepsis and meningitis in immune deficient patients and abortion in pregnant women (1, 7, 8). *L. monocytogenes* is a food-borne pathogen, its incidence and growth in foods, contribute to outbreaks of listeriosis (9). The presence of the organism in pregnancy, may be asymptomatic or associated with vaginal infection in women and represents many risks such as stillbirth, abortion or, serious consequences in neonate, including early onset with bacteremia, pneumonia, conjunctivitis and skin lesions, or late onset with meningitis (10, 11).

There is strongly association between antigenic compounds of *Listeria* spp. and their pathogenicity, which also causes heterogeneity in virulence of *L. monocytogenes* between food and clinical strains (12). The pathophysiology of Listerial infection is associated with adherence and invasion of the gastrointestinal epithelium by the organism, which is mainly mediated by internalin A (*InlA*), and internalin B (*InlB*) (13). It has been proved that *InlA* fragment contributes to the invasiveness of *L. monocytogenes* and could differentiate potentially invasive bacteria from noninvasive strains (14). Moreover, pore-forming hemolysin listeriolysin O (*hlyA*), is required for virulence of *L. monocytogenes* and is only found in virulent strains (15).

Due to the widespread distribution of *L. monocytogenes* in environmental and animal sources and serious clinical complications in human, this study was aimed to isolate *L. monocytogenes* from different parts of Karun River as the largest water source in Khuzestan province, Iran, and also from clinical specimens by culture and PCR methods. Moreover, the presence of *hlyA* and *inlA* virulence genes and the relationship between environmental and clinical samples were also investigated by PCR-Restriction Fragment Length Polymorphism (RFLP) techniques.

## MATERIALS AND METHODS

### Sampling and isolation of *L. monocytogenes.*

A total of 180 water samples from different parts of Karun River in Khuzestan province, Iran, were collected from March to May 2016. The initial proposal of the work was approved by the Institutional Review Board (IRB) and Ethics Committee of the Ahvaz Jundishapur University of Medical Sciences, Iran, and necessary permission was received for the work. Isolation of *L. monocytogenes* from water samples was performed according to MFHBP-30 method (16), with some modifications. In brief, 100 ml of water was filtered through 0.45 μm pore size filters. The water filters were then transferred into 9 ml of Listeria Enrichment Broth (LEB) (Merck, Germany) and incubated at 30°C for 48 hours. One mL of grown LEB was transferred into a bottle containing 9 mL of Fraser Listeria Selective Enrichment Broth (Merck, Germany), and incubated at 37°C for 24 hours. From Fraser grown medium, 50 μl was streaked onto Listeria Chromogenic Agar (Merck, Germany), and incubated at 37°C for 24 hours. The blue colonies with white halos were selected for microbiological tests.

The clinical samples were included 80 vaginal swabs and 80 stool specimens, which were collected from women with at least two abortions. Both clinical samples were inoculated into the Listeria Enrichment Broth and processed in the same manner described for water samples.

Identification of the *L. monocytogenes* was done according to the standard microbiological tests such as catalase, oxidase, β haemolysis, motility test at 25°C, the CAMP assay, and acid production from xylose and rhamnose (17).

### DNA extraction and PCR amplification.

DNAs were extracted from the colonies grown on Chromogenic Agar medium, by High pure PCR Template Preparation Kit (Roche Co., Germany). For amplification of *hlyA* gene, the set of primers of (F, 5′-GAATGTAAACTTCGGCG-CAATCAG-3′) and (R, 5′-GCCGTCGATGATTTGAACTTCATC-3′), were used which amplify a 388bp fragment of the target gene (18). PCR amplification was performed in a final volume of 25 μl containing 10× PCR buffer, 1.5 mM MgCl_2_, 10 mM dNTPs, 0.5 mM of each primer, 1.5U *Taq* polymerase and 5 μl of template DNA. All the reagents were purchased from Qiagen, Hilden, Germany. Amplification was performed on a thermocycler nexus gradient (Eppendorf), and the cycling program consisted of initial denaturation at 95°C for 5 min, followed by 30 cycles of denaturation at 95°C for 45 s, annealing at 56°C for 45 s, extension at 72°C for 60 s, and a final extension at 72°C for 7 min. For amplification of *inlA* gene, a set of primers (seq 01: 5′ AATCTAGCACCACTGTCGGG 3′) and (seq 02: 5′TGTGACCTTCTTTTACGGGC 3′) was used to amplify a 733 bp fragment of the target gene (19). The reaction volume and cycling condition were the same as described for the *hlyA* gene, with the exception of annealing temperature of 52°C. *L. monocytogenes* ATCC-764 and *Escherichia coli* ATCC 25922 were used as positive and negative controls respectively, and were included in each PCR run. The PCR products were analyzed by gel electrophoresis on 2% agarose gel (w/vol.) containing 0.5 mg/mL ethidium bromide (Qiagen, Hilden, Germany). The results were recorded using the gel documentation system (Protein Simple, San Jose, CA, USA). A 100-bp DNA ladder was used as a size marker (Roche, Germany).

### RFLP analysis of *inlA*.

A 733-bp fragment of *inlA* gene was used for investigation of polymorphism as described by Rousseaux et al. (19), Two restriction endonucleases of *AluI* and *Tsp509I* were used for digestion of *inlA* amplification products. Briefly, 10 μl of fresh PCR product was added to 18 μl nuclease free water, 2 μl 10X buffer tango and 1 μl restriction enzyme, mixed gently and incubated at 37°C for 16 hours, followed by a step of enzyme inactivation at 65°C for 20 min. PCR-RFLP fragments were separated by electrophoresis on a 3.5% agarose gel.

For data analysis, the descriptive statistics, Chi-square and logistics regression tests were performed in SPSS version 16.00.

## RESULTS

**Fig. 1.**
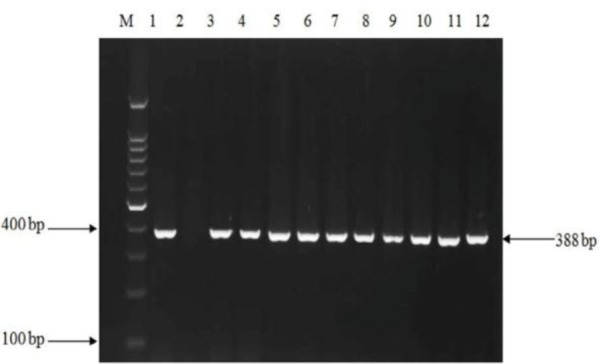
PCR amplification of the *hlyA* gene in *L. monocytogenes* isolates. M, 100 bp DNA size marker; lane 1, positive control *L. monocytogenes* ATCC (388 bp); Lane 2, negative control; lanes 3 to 7, isolates from water samples; lanes 8 to 10, isolates from vaginal swabs; and lanes 10 to 12, isolates from fecal samples.

**Fig. 2.**
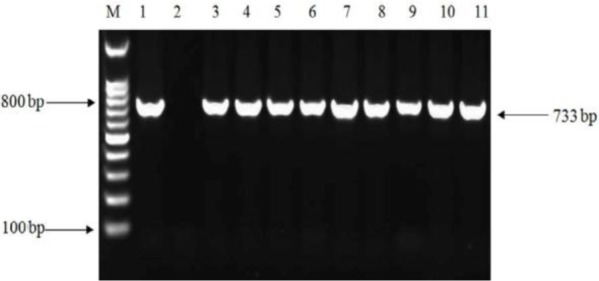
PCR amplification of *inlA* gene in *L. monocytogenes* isolates. M, 100 bp DNA size marker; lane 1, positive control *L. monocytogenes* ATCC (733 bp); lane 2, negative control; lanes 3 to 6, isolates from water samples; lanes 7 to 9, isolates from vaginal swabs; and lanes 10 to 11, isolates from fecal samples.

From total tested water samples, 30 (16.7%) had a well grown on chromogenic agar, while of 160 samples of vaginal swabs and feces, 9 (11.3%) and 6 (7.5%), were positive in culture respectively. For 45 samples with an initial positive culture, diagnostic microbiological tests were performed, and all isolates were identified as *L. monocytogenes* on the basis of β-hemolysis, catalase activity, motility at 25°C, positive CAMP test, acid production from rhamnosus, and xylose and negative oxidase test. On performing PCR amplification, all 45 samples had a positive result for both *hlyA* and *inlA* genes (Figs. 1 and 2).

The restriction enzyme analysis of PCR products using *AluI* and *Tsp509I*, generated two profiles with 8 to 10 bands ranging in size from 15 to 210 bp according to Rousseaux et al. (19), For *AluI* digestion, the majority of water and clinical isolates were grouped in profile 2 as 70%, 77.7% and 88.3% for water, vaginal and fecal isolates respectively (Table 1).

**Table 1.** RFLP profiles of water and clinical isolates digested with *AluI* and *Tsp5091* restriction enzymes in the present study

**Restriction profiles**	**Water isolates No (%)**	**Vaginal isolates No (%)**	**Fecal isolates No (%)**
*AluI*			
Profile 2	21 (70)	7 (77.7)	5 (83.4)
Profile 3	9 (30)	2 (22.3)	1 (16.6)
*Tsp5091*			
Profile 2	23 (76.7)	6 (66.7)	4 (66.7)
Profile 3	7 (23.3)	3 (33.3)	2 (33.3)

**Fig. 3.**
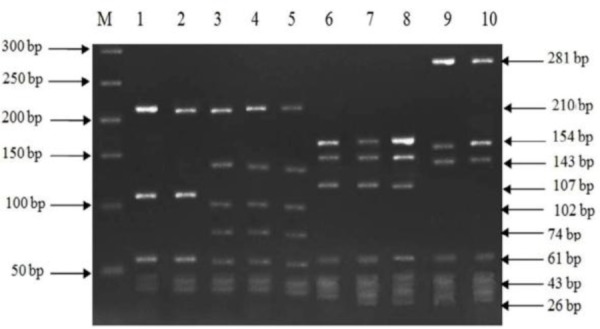
RFLP profiles of *L. Monocytogenes* isolates after digestion with *AluI* and *Tsp5091* enzymes. M, 50 bp molecular size marker; *AluI* digestion results: lanes 1 and 2, profile 2; lanes 3 to 5, profile 3. *Tsp5091* digestion results: lanes 6 to 8, profile 2; lanes 9 and 10, profile 3.

Digestion by *Tsp509I* enzyme generated similar grouping, as most of the water and clinical isolates were classified in profile 2 (Fig. 3).

## DISCUSSION

*Listeria monocytogenes* is isolated from a diversity of several sources, but municipal sewage sludge and water are mainly important in the transmission of this bacterium. *L. monocytogenes* is colonized in the gastrointestinal tract and vagina, therefore, it can be a potential pathogen and a public health concern. *L. monocytogenes* infection in pregnant women, increases the risk of abortion, and also it can be transmitted from mother to fetus through the birth canal and create serious hazards for the neonate (1, 10, 16).

In this study, due to the importance of water in the transmission of *L. monocytogenes*, water samples from different parts of Karun River were microbiologically screened. Karun River is a large supplier of water for Khuzestan province, so any serious microbial pollution of this river affecting agricultural farms, and people who are living in villages near the river, where they often use water from river directly for drinking.

From total 180 examined water samples, 30 (16.7%), were positive for *L. monocytogenes*. There are different reports of isolation of *L. monocytogenes* from rivers, for instance in a study a rate of isolation of 54.5% has also been reported which was higher than our findings (20).

In addition to water, clinical samples of vaginal swabs and feces were also studied in the present study. Samples of vaginal swabs were collected from women with at least two abortions. Of 80 vaginal samples, 9 (11.3%) were positive for *L. monocytogenes*. This rate was lower in comparison to the rate of 16.6% in the study of Eslami et al. (21), and 14.2% reported by Pournajaf et al., both from Iran (22). In both latter studies, the reason for the difference is the different geographic locations studied. In the current study and other similar studies, the prevalence of *L. monocytogenes* is reported in women with abortion history. As other investigators statement, during pregnancy, due to the increase in the amount of progesterone, cellular immunity is weakened and this makes the pregnant women mainly susceptible to intracellular microorganisms like *L. monocytogenes* (23). In this study, 80 samples of feces were collected which 6 (7.5%) samples had a positive culture. This finding was in concordant to the rate of 8.5% reported by Pournajaf et al. (22), and 6% in the study of Lyautey et al., which in both studies *L. monocytogenes* was isolated from fecal samples (24). In disagreement to our study, lower rate of 0.12% *L. monocytogenes* from fecal samples were reported by Sauders et al. in four large metropolitan areas of New York state (25), which the difference in geographical areas may be the reason for this difference.

According to the results from PCR assay, all the positive 45 samples detected by phenotypic tests, including 30 water samples, 9 vaginal swabs and 6 fecal samples had a positive result for both *hlyA* and *inlA* genes. In some previous studies, the work was only focused on water samples and no clinical specimens were included to be able to find out the probable correlation between *L. monocytogenes* isolates from water and those from clinical source in view of genetic similarities (5, 16, 20). In this work, for investigation of a probable link between strains isolated from water sources and clinical specimens, RFLP analysis on *inlA* were performed. Using endonucleases *AluI* and *Tsp509I*, two profiles were generated. The majority of water, vaginal and fecal isolates were classified in profile 2 for both restriction enzyme tested and this demonstrates that water of Karun River, could be the potential source of *L. monocytogenes* to human infection in the present study. In the study of Rousseaux et al. which amplified *inlA* fragment was digested by endonucleases *AluI* and *Tsp509I*, five profiles were generated for *AluI*, and three profiles for *Tsp509I* endonucleases (19).

In the current study, 45 *L. monocytogenes* isolates were cultured from tested water and clinical samples and confirmed by phenotypic and molecular tests. The majority of the isolates were classified in profile 2 as RFLP analysis revealed, showing the clinical isolates were mainly originated from water. Due to the invasive nature of *L. monocytogenes*, and the various diseases that this bacterium causes in animals and humans especially pregnant women, further studies on *L. monocytogenes* appear to be necessary.
